# Author Correction: Molecular architecture of the multifunctional collagen lysyl hydroxylase and glycosyltransferase LH3

**DOI:** 10.1038/s41467-018-06481-x

**Published:** 2018-09-20

**Authors:** Luigi Scietti, Antonella Chiapparino, Francesca De Giorgi, Marco Fumagalli, Lela Khoriauli, Solomon Nergadze, Shibom Basu, Vincent Olieric, Lucia Cucca, Blerida Banushi, Antonella Profumo, Elena Giulotto, Paul Gissen, Federico Forneris

**Affiliations:** 1 The Armenise-Harvard Laboratory of Structural Biology, Department of Biology and Biotechnology, University of Pavia, Via Ferrata 9/A, 27100 Pavia, Italy; 2 Laboratory of Biochemistry, Department of Biology and Biotechnology, University of Pavia, Via Taramelli 3/B, 27100 Pavia, Italy; 3 Laboratory of Molecular Biology, Department of Biology and Biotechnology, University of Pavia, Via Ferrata 9/A, 27100 Pavia, Italy; 4 Swiss Light Source, Paul Scherrer Institut, Villigen, 5232 Switzerland; 5 Laboratory of Analytical Chemistry, Department of Chemistry, University of Pavia, Via Taramelli 12, 27100 Pavia, Italy; 6 MRC Laboratory for Molecular Cell Biology, University College London, WC1E 6BT London, UK; 7 UCL Great Ormond Street Institute of Child Health, 30 Guilford Street, WC1N 1EH London, UK; 8 Present Address: Translational Research Institute, The University of Queensland Diamantina Institute, Princess Alexandra Hospital, 37 Kent Street, Brisbane, Australia

Correction to: *Nature Communications*; 10.1038/s41467-018-05631-5; published online 08 August 2018

The previously published version of this Article contained an error in Fig. 3. In Fig. 3a, the residues His667 and Asp669 were incorrectly labelled as His627 and Asp629. The error has been corrected in both the PDF and HTML versions of the Article.

