# Supplementary material for: Molecular architecture of the multifunctional collagen lysyl hydroxylase and glycosyltransferase LH3
Source: Nat Commun. 2018 Aug 8;9:3163. doi: 10.1038/s41467-018-05631-5 (PMC6082870; doi:10.1038/s41467-018-05631-5)
Supplement: Supplementary file 1 — Supplementary Information [file 41467_2018_5631_MOESM1_ESM.pdf]

L. Scietti *et al.*

Molecular architecture of the multifunctional collagen lysyl hydroxylase and glycosyltransferase LH3

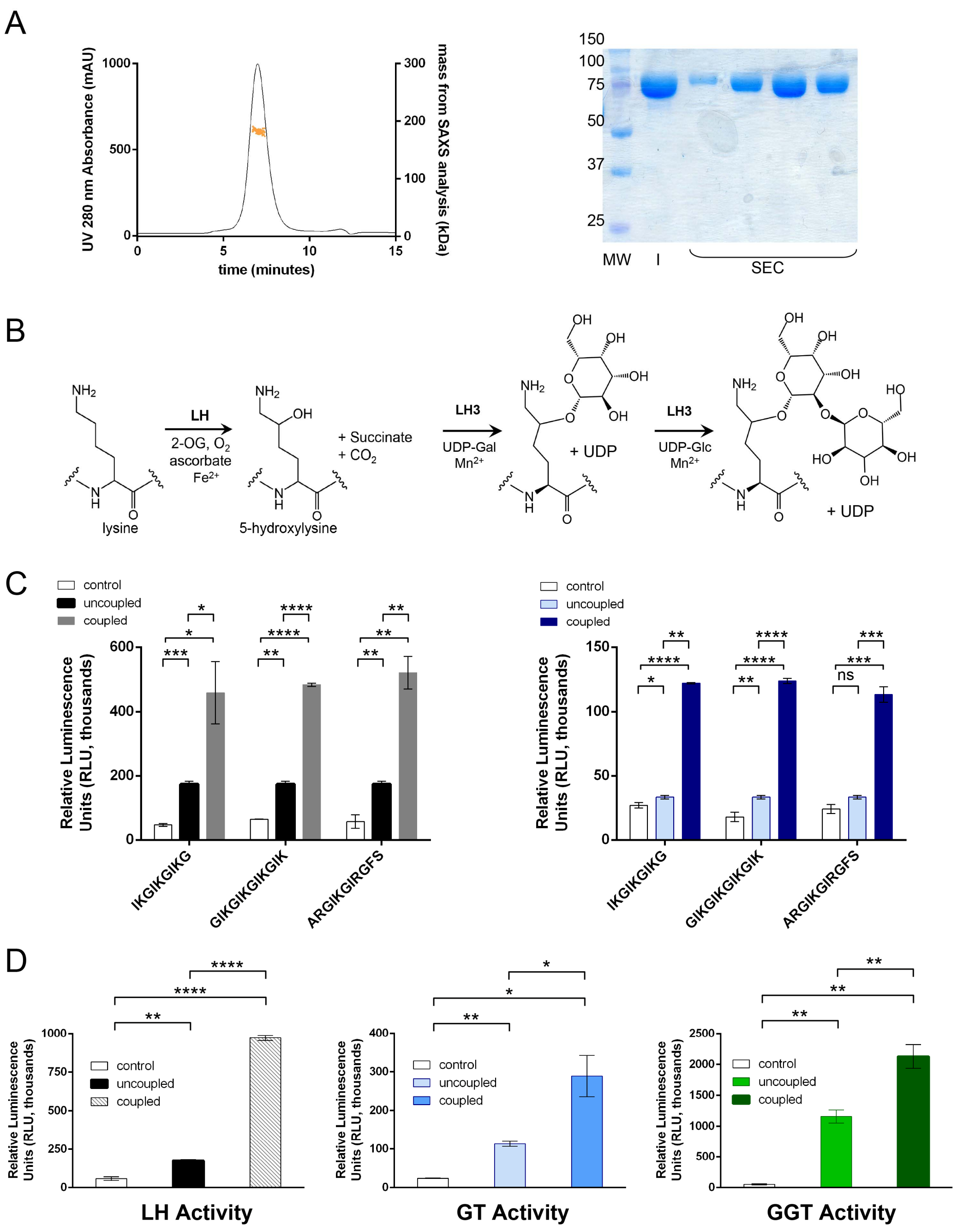

Supplementary Figure 1: recombinant production and biochemical characterization of full-length human LH3. (A) LH3 elutes as a dimer in size exclusion chromatography experiments. Shown is a typical SEC-SAXS chromatogram illustrating the molar mass derived from SAXS (orange dots) associated to the elution peak, corresponding to ~200 kDa. The associated SDS-PAGE shows, from left to right: molecular weight markers (MW), LH3 sample prior to SEC analysis (I), and four fractions from the single peak observed in the chromatogram. (B) Schematic of the reactions catalyzed by LH3. (C) Evaluation of LH3 lysyl hydroxylase (LH, left) and glycosyltransferase (GT, right) activities on synthetic peptides using luminescence-based enzymatic assays (see methods). Control experiments were carried out without enzyme in the reaction mix. The LH assays highlights significant (~25%) uncoupling. Error bars represent standard deviations from average of triplicate independent experiments. Statistical evaluations based on pair sample comparisons using Student's t-test. ns, non-significant; *, P-value < 0.05; **, P-value < 0.01; ***, P-value < 0.001; ****, P-value < 0.0001. (D) Evaluation of LH3 lysyl hydroxylase (left), galactosyltransferases (middle) and glucosyltransferase activities on gelatin using luminescence-based enzymatic assays (see methods). Error bars and statistical analyses as in panel (C).

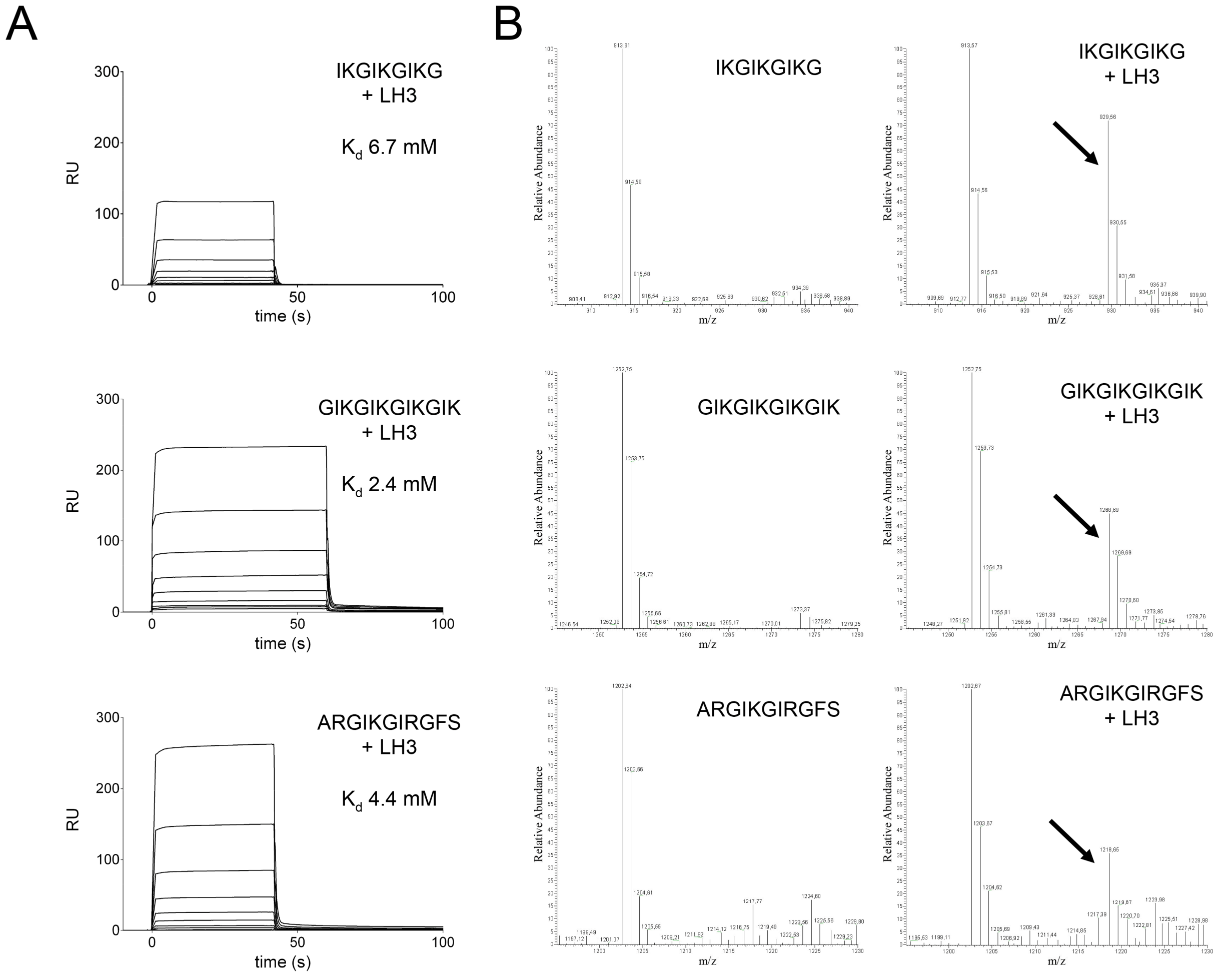

Supplementary Figure 2: evaluation of LH3 reactivity of synthetic peptides. (A) SPR measurement of binding of three synthetic peptides conventionally used to assess LH3 enzymatic activity on immobilized LH3. Concentration ranges were between 8 μM and 2 mM in all experiments. K_d values were calculated at the steady-state using the Biacore T200 evaluation software (GE Healthcare). (B) MS spectra of the three synthetic peptides as in panel (A) before (left) and after (right) LH3 treatment. These spectra are characterized by a series of sequential peaks, corresponding to a content of one, two or three ^{13}C atoms. After enzyme treatment, additional peaks corresponding to +16 Da, associated to lysine hydroxylation, are visible as highlighted by the arrow in the right panels.

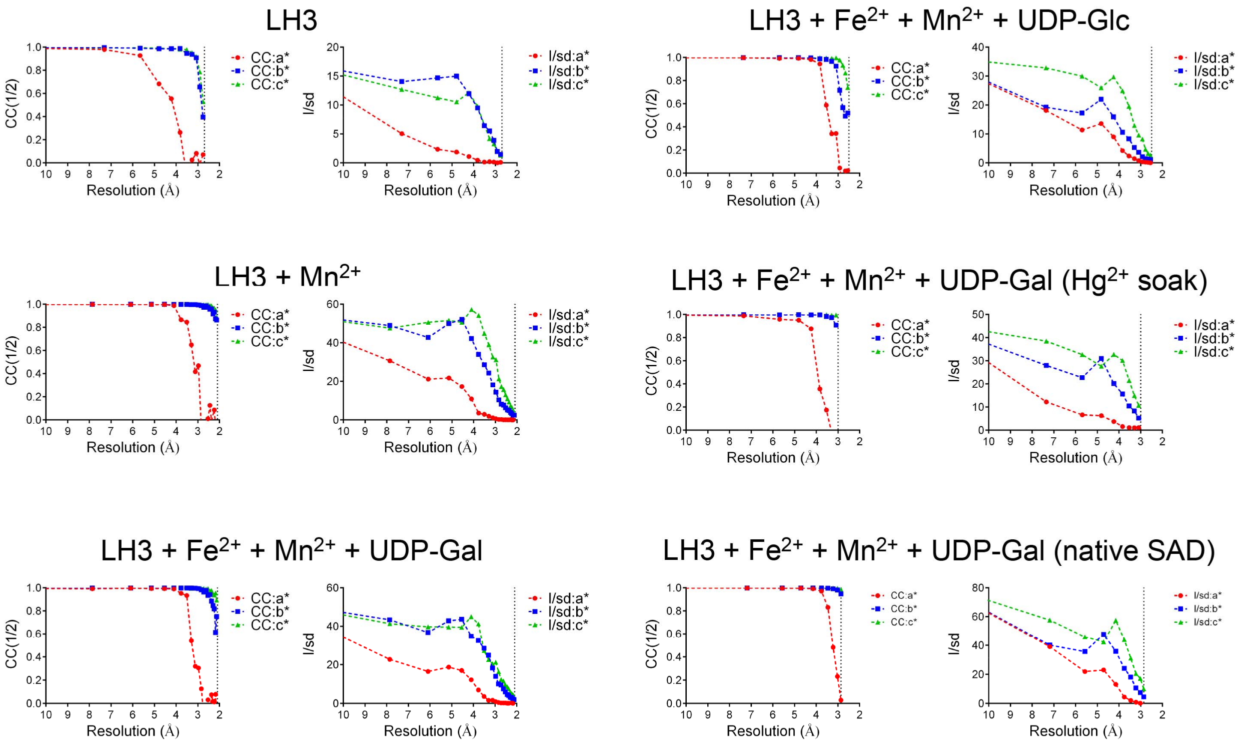

Supplementary Figure 3: LH3 crystals diffract anisotropically. The graphs show AIMLESS[1] analysis of CC1/2 and I/σ in the three directions of reciprocal space as function of resolution for each of the datasets described in Supplementary Table 1. For native-SAD measurement, we collected multi-orientation multiple datasets (n x 360deg) on each crystal, which were then merged and processed using AutoPROC[2]. The anisotropy referring to one of the multiple crystals collected and merged is shown. The anisotropy values reported below are the maximum resolutions in each direction of reciprocal space from scaling of multiple datasets on each individual crystal during the AutoPROC run. Crystal 1 (7 datasets): a* 3.583, b* 2.298, c* 2.194; crystal 2 (15 datasets): a* 3.281, b* 2.352; c* 2.121; crystal 3 (16 datasets): a* 3.688, b* 2.880, c* 2.605; crystal 4 (2 datasets): a* 5.073, b*, 3.581, c* 2.617; crystal 5 (9 datasets): a* 4.148, b* 2.392, c* 2.201; crystal 6 (8 datasets): a* 4.499, b* 2.693, c* 2.414.

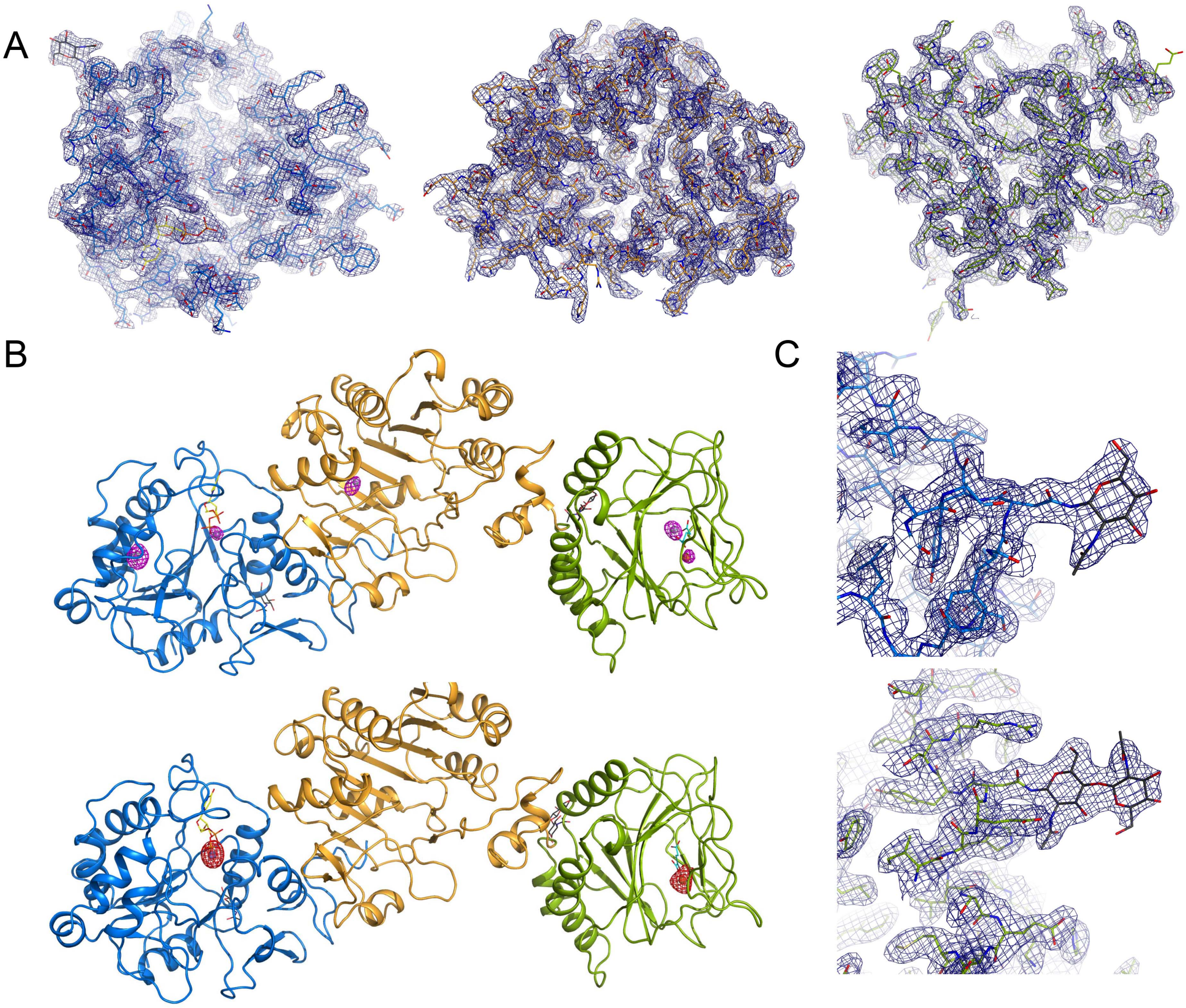

Supplementary Figure 4: overview of the quality of the experimental structural data. (A) Refined $2F_o$-F_c electron density maps (contour level 1.5 σ) covering the GT (left), AC (center) and LH domains (right) of LH3. (B) Anomalous electron density maps (contour level 8 σ, top panel) computed from the dataset obtained from Hg^{2+} soaking highlight the presence of three very strong peaks, attributed to Hg^{2+}, and less intense peaks corresponding to the positions of Mn^{2+} and Fe^{2+} ions. These metal ions were also confirmed through calculation of anomalous maps obtained in long wavelength diffraction datasets from native crystals (contour level 6 σ, bottom panel). (C) Details of the refined $2F_o$-F_c electron density (contour level 1.5 σ) protruding from the LH3 polypeptide chain near Asn63 (top panel) and Asn548 (bottom panel), showing N-linked glycosylations.

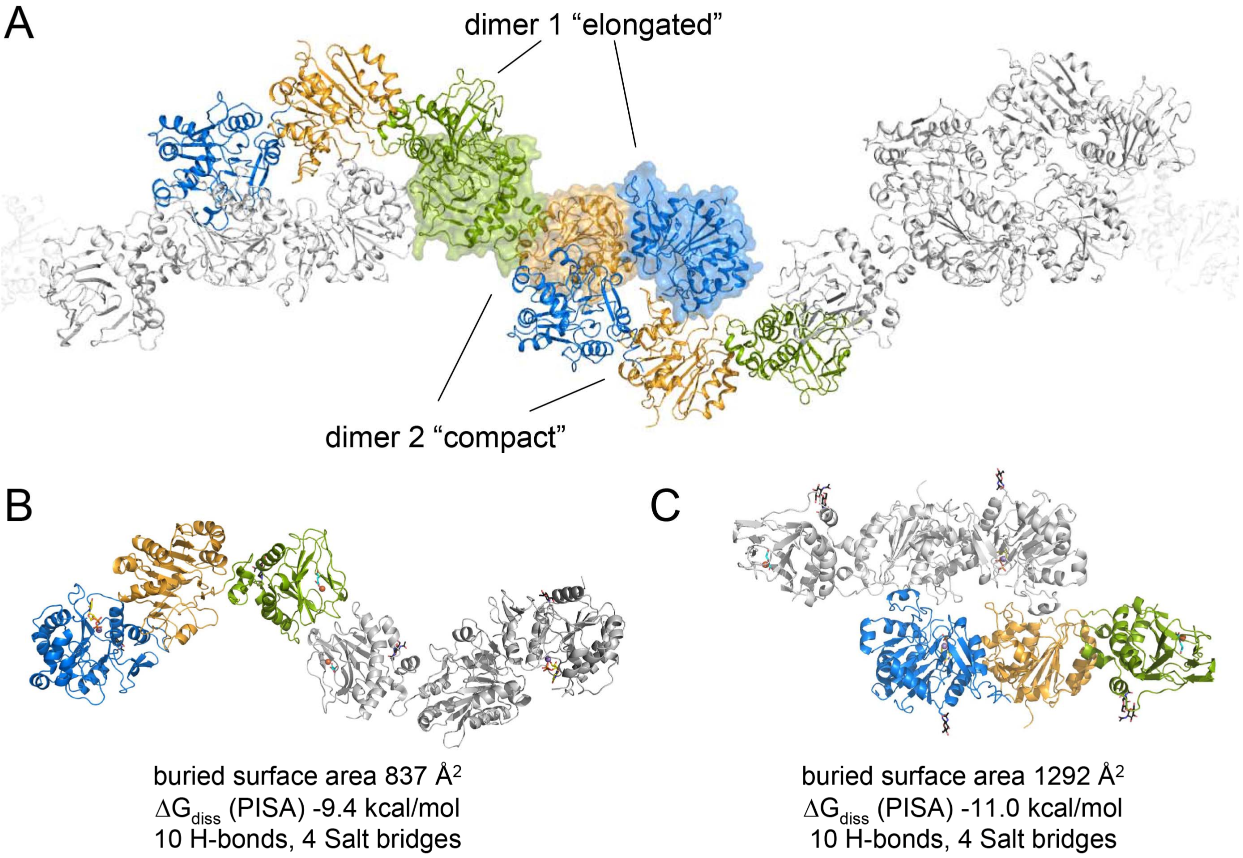

Supplementary Figure 5: dimer interfaces observed in the LH3 crystal packing. (A) In LH3 crystal structures, only one molecule (shown as semi-transparent surface) is present in the asymmetric unit. However, two dimeric assemblies can be detected in the crystal packing: a tail-to-tail elongated dimer, and a more compact dimer that exposes the C-terminal domains. (B) Cartoon representation of the elongated tail-to-tail LH3 dimer, with indication of the features found in the dimer interface as assessed by PISA[3]. (C) Cartoon representation of the compact LH3 dimer, with indication of the features found in the dimer interface as assessed by PISA[3]. For clarity, symmetry-related molecules are shown as white cartoons. Domain coloring as in Figure 1.

A

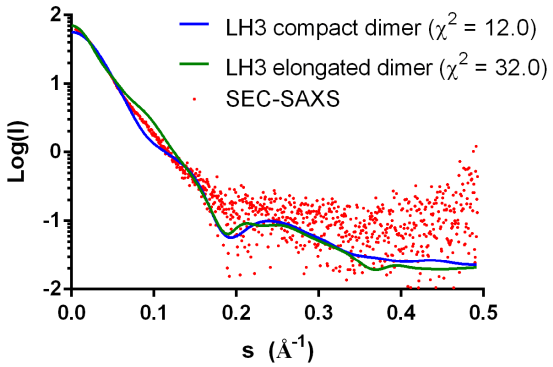

B

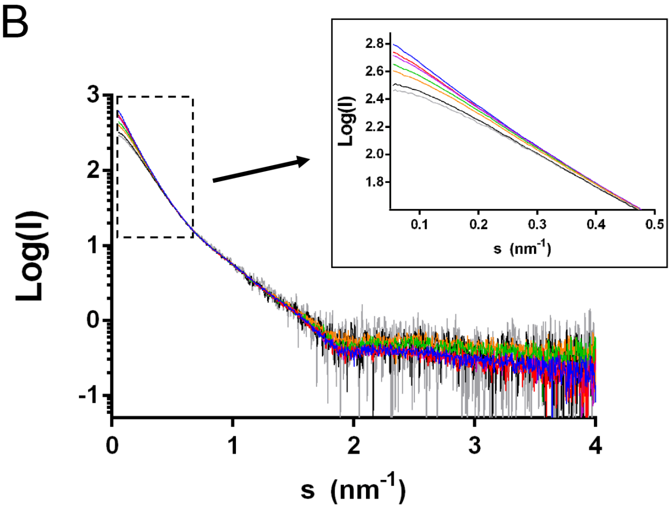

Concentration	Rg (nm)	Dmax (nm)
0.6 mg/mL	6.9	23.5
1.1 mg/mL	7.3	30.9
2.0 mg/mL	8.3	31.5
2.5 mg/mL	8.9	36.1
3.4 mg/mL	9.5	40.9
4.2 mg/mL	10.1	47.1
9.0 mg/mL	10.9	48.6

Supplementary Figure 6: characterization of LH3 in solution using SAXS. (A) Comparison of the crystallographic LH3 dimers with experimental SEC-SAXS data using CRYSOL[4] shows that none of the crystal-derived assemblies can unambiguously match the state adopted by the protein in solution. (B) Batch SAXS measurements at different enzyme concentration reveal concentration-dependent oligomerization, with large variations in the radius of gyration (R_g) and maximum dimension (D_{max}) values obtained from Guinier and pair distance distribution analyses in PRIMUS[5].

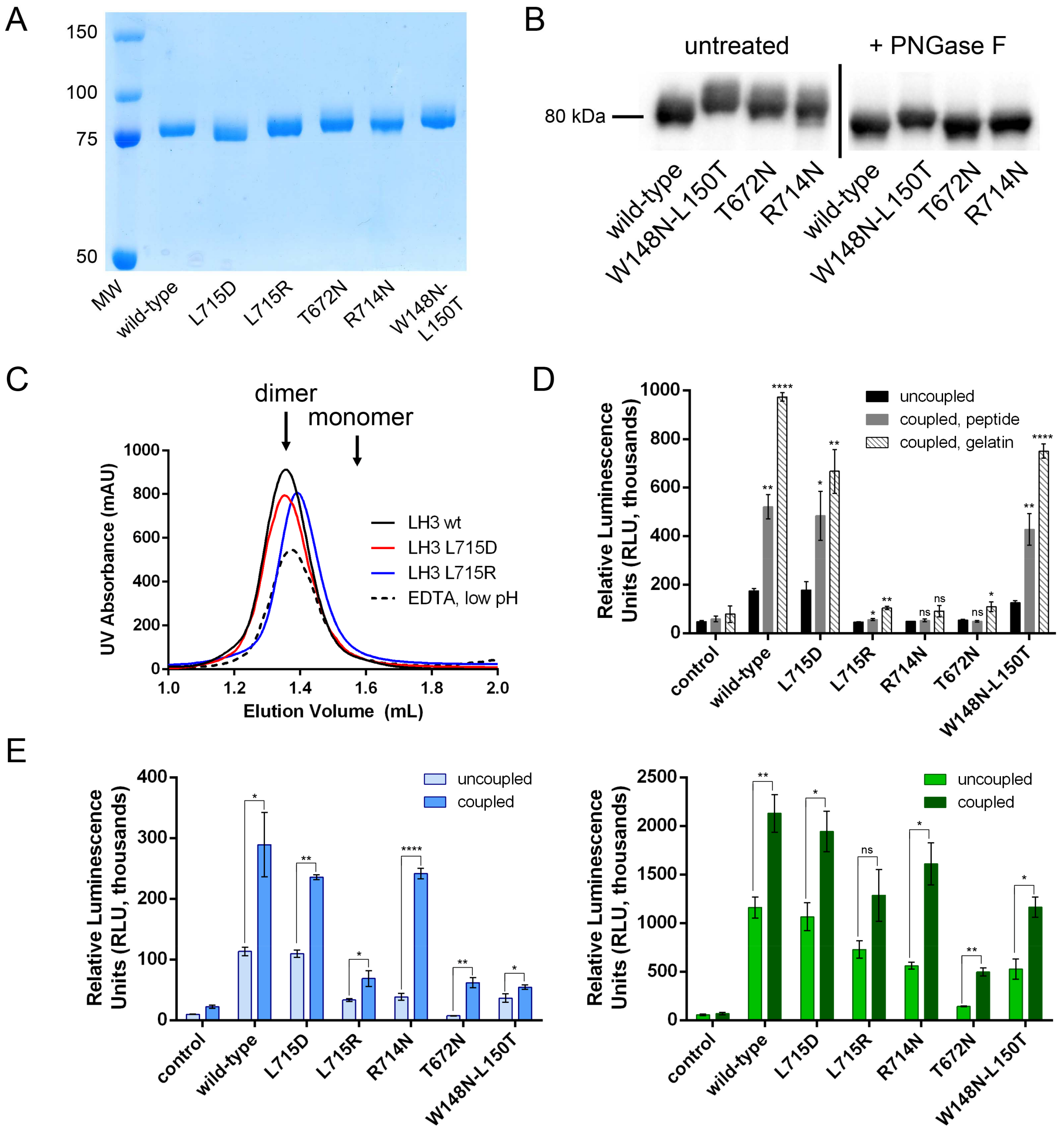

Supplementary Figure 7: mutagenesis to probe the LH3 dimeric assembly. (A) SDS-PAGE analysis of purified LH3 mutants designed to disrupt putative binding interfaces. Mutants T672N, R714N, and W148N-L150T show smeared bands at slightly higher molecular weight compared to wild-type LH3, in line with the introduction of an additional glycosylation. (B) Effective introduction of additional glycosylation was validated using western blot analysis with an anti-LH3 antibody on untreated and PNGase F-treated LH3 mutants. (C) Mutants L715D and L715R do not disrupt the LH3 dimer, as assessed by size-exclusion chromatography. The dashed line in the chromatogram shows that attempts to remove Fe^{2+} from wild-type LH3 as done by Guo et al.[6] did not alter the enzyme's oligomeric state. (D) Evaluation of the LH enzymatic activity of the mutants designed to probe the LH3 dimeric assembly using synthetic peptides (grey) or gelatin (striped white) as substrates. Error bars and statistical evaluations as in Figure 2 and Supplementary Fig. 1. (E) Evaluation of the GT (left) and GGT (right) enzymatic activities of the same mutants using gelatin as substrate. Error bars and statistical evaluations as in Figure 2 and Supplementary Fig. 1.

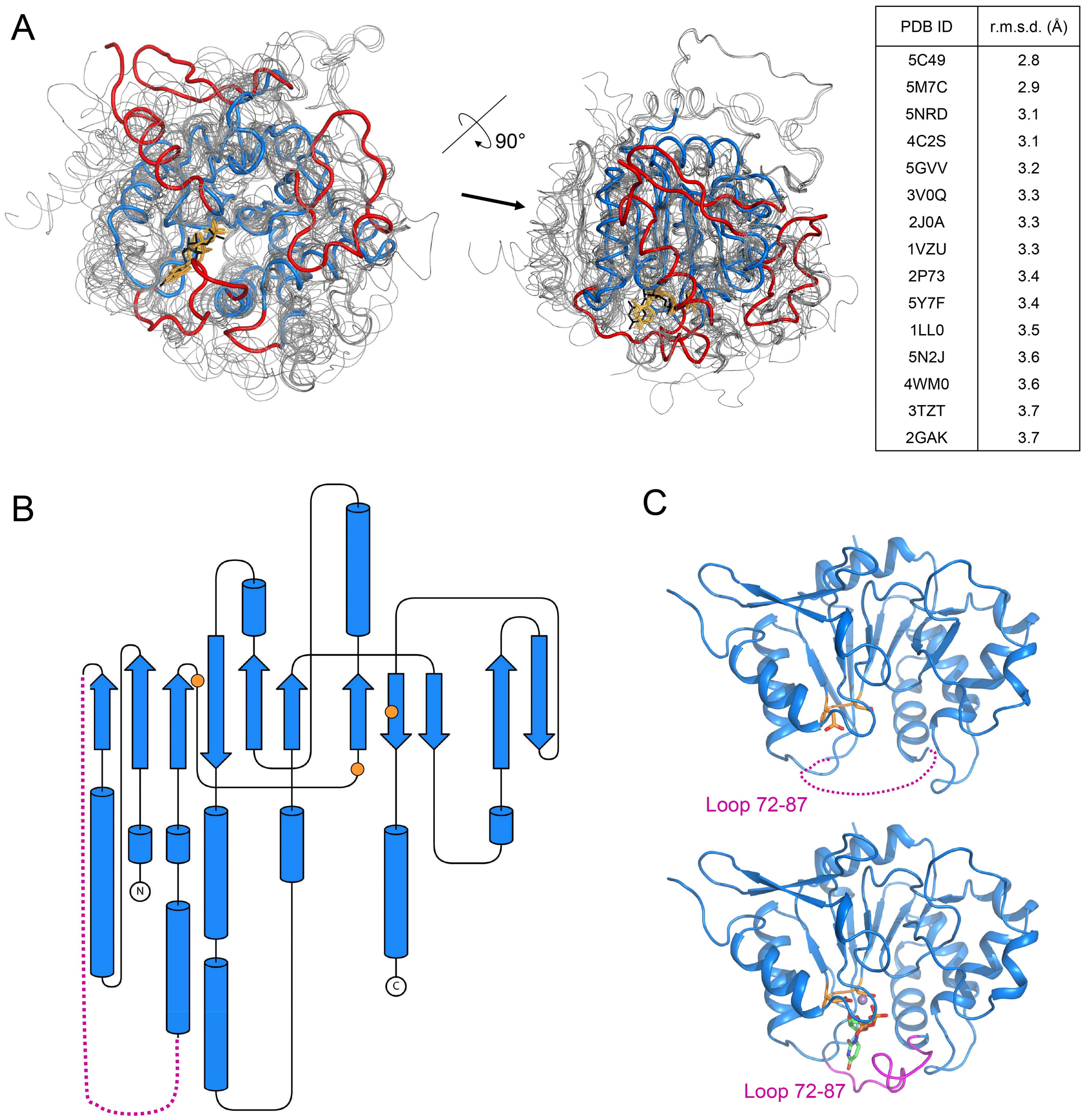

PDB ID	r.m.s.d. (Å)
5C49	2.8
5M7C	2.9
5NRD	3.1
4C2S	3.1
5GVV	3.2
3V0Q	3.3
2J0A	3.3
1VZU	3.3
2P73	3.4
5Y7F	3.4
1LL0	3.5
5N2J	3.6
4WM0	3.6
3TZT	3.7
2GAK	3.7

Supplementary Figure 8: unique structural features observed in the N-terminal GT domain of LH3. (A) Superposition of the LH3 GT domain (thick ribbon, blue) with glycosyltransferase structures identified by DALI[7] and PDBeFold[8] show that, besides the classical Rossmann fold architecture, this domain contains numerous unique features, as highlighted by the high r.s.m.d. values obtained from superpositions (listed in the table on the right). Uniquely shaped loops defining the LH3 GT catalytic site (thick ribbon) are shown in red. To facilitate visualization, identified glycosyltransferase domains (thin ribbons, grey) are shown in two different orientations. The UDP molecule found in the LH3 GT domain (black sticks) superimposes well with other UDP molecules trapped in some of the structures used for comparisons (orange sticks). The arrow indicates the conserved α-β hairpin found in glycosyltransferases that is not present in the LH3 GT domain. (B) Topology diagram of the LH3 GT domain. Colors identify the N- and the C- subunits characterizing this domain, separated by the flexible loop defined by residues 72-87 (dashed line in magenta). The position of the two Asp and the His residues involved in Mn^{2+} coordination are shown with orange circles. This image was created with TOPDRAW[9]. (C) Cartoon representation of the ligand-free (top) and ligand-bound states of the LH3 GT domains, colors as in (B). Residues involved in Mn^{2+} coordination are shown with orange sticks.

```
                   33          43          53          63          73
Human LH3          PVNPEKLLVI  TVATAETEGY  LRFLRSAEFF  NYTVRTLGLG  EEWRGGDVAR
Human LH1          AKPEDNLLVL  TVATKETEGF  RRFKRSAQFF  NYKIQALGLG  EDWNVEKGT-
Human LH2          SIPTDKLLVI  TVATKESDGF  HRFMQSAKYF  NYTVKVLGQG  EEWRGGDGIN
consensus          .   ::***:  **** *::*:   ** .**::*  **.:..** *  *:*.  .

                   83          93          103         113         123
Human LH3          TVGGGQKVRW  LKKEMEKYAD  REDMIIMFVD  SYDVILAGSP  TELLKKFVQS
Human LH1          SAGGGQKVRL  LKKALEKHAD  KEDLVILFAD  SYDVLFASGP  RELLKKFRQA
Human LH2          SIGGGQKVRL  MKEVMEHYAD  QDDLVVMFTE  CFDVIFAGGP  EEVLKKFQKA
consensus          : *******   :*: :*::**  .:*::::*.:  .:**::*..*  *:**** ::

                   133         143         153         163         173
Human LH3          GSRLLFSAES  FCWPEWGLAE  QYPEVGTGKR  FLNSGGFIGF  ATTIHQIVRQ
Human LH1          RSQVVFSAEE  LIYPDRRLET  KYPVVSDGKR  FLGSGGFIGY  APNLSKLVAE
Human LH2          NHKVVFAADG  ILWPDKRLAD  KYPVVHIGKR  YLNSGGFIGY  APYVNRIVQQ
consensus            .::*:*:   : :*:. *    :** *  ***  :*.******:  *. : .:* :

                   183         193         203         213         223
Human LH3          WKYKDDDDDQ  LFYTRLYLDP  GLREKLSLNL  DHKSRIFQNL  NGALDEVVLK
Human LH1          WEGQDSDSDQ  LFYTKIFLDP  EKREQINITL  DHRCRIFQNL  DGALDEVVLK
Human LH2          WNLQDNDDDQ  LFYTKVYIDP  LKREAINITL  DHKCKIFQTL  NGAVDEVVLK
consensus          *: :*.*.**  ****.:::**    ** :.:.*  **...***.*  :**:******

                   233         243         253         263         273
Human LH3          FDRNRVRIRN  VAYDTLPIVV  HGNGPTKLQL  NYLGNYVPNG  WTPEGGCGFC
Human LH1          FEMGHVRARN  LAYDTLPVLI  HGNGPTKLQL  NYLGNYIPRF  WTFETGCTVC
Human LH2          FENGKARAKN  TFYETLPVAI  NGNGPTKILL  NYFGNYVPNS  WTQDNGCTLC
consensus          *: ...* .*    *:***: :  :******: *  **:***:*.   ** : ** .*

                   283
Human LH3          NQDRRTLPGG
Human LH1          DEGLRSLKGI
Human LH2          EFDTVDLSAV
consensus          : .   * .
```

Supplementary Figure 9: sequence alignment between GT domains of human LH isoforms. The sequences of the unique loops shaping the GT catalytic site of LH3 are shown in red. The region highlighted in violet contains the flexible loop that interacts with donor substrates and stabilizes UDP binding. Other aminoacids involved in direct interactions with donor substrate are shown with blue triangles. The poly-Asp region proximate to the catalytic site is shown in dark red. Residues involved in Mn^{2+} coordination are shown with orange triangles. The pathogenic mutation N223S is shown in magenta. Residues Trp145 and Trp148 subject to conformational rearrangements upon UDP-donor substrate binding are shown with green triangles. The flexible loop linking the GT and AC domains is shown in light blue. Disulfide bonds are shown with connecting lines. The "consensus" line, derived from the alignment software, indicates conservation of sequence identity or similarity as follows: "*" indicates a fully conserved residue; ":" indicates conservation between groups of strongly similar properties; "." indicates conservation between groups of weakly similar properties.

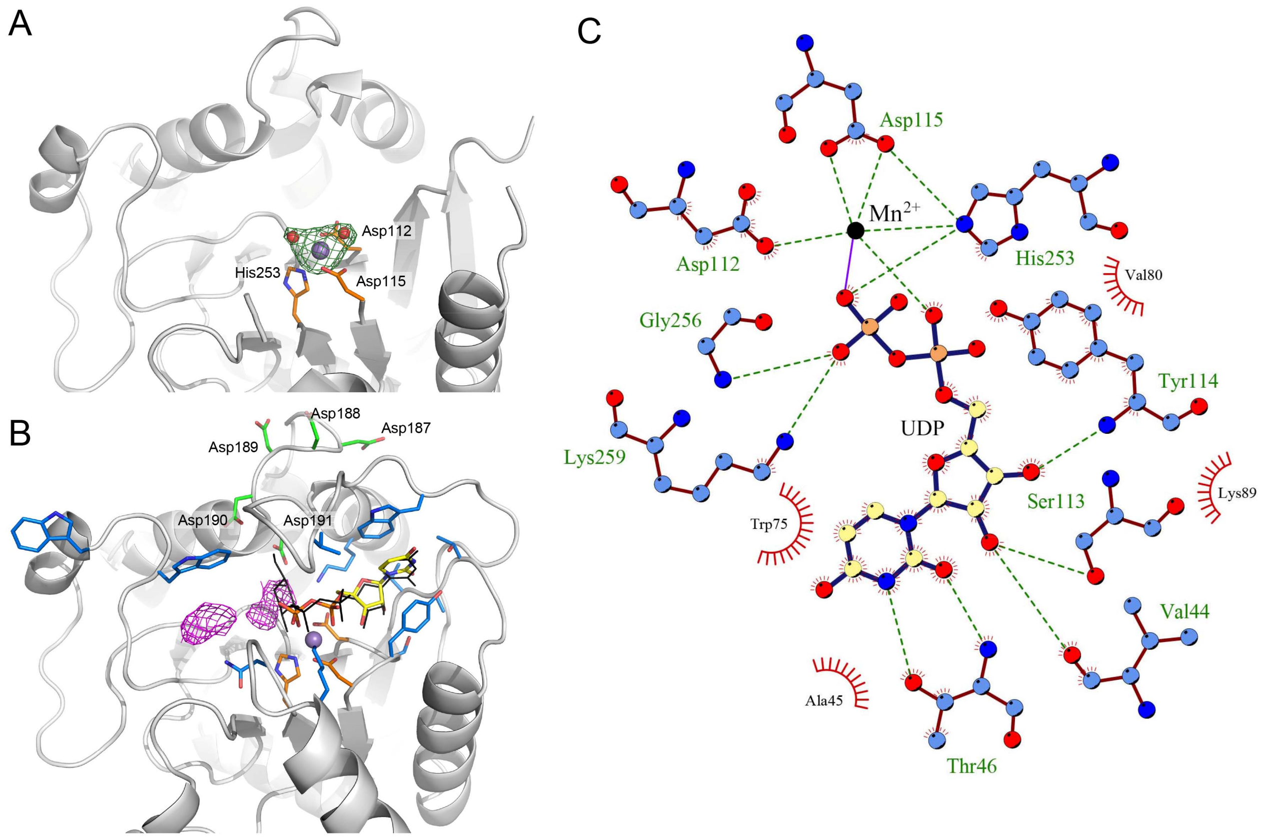

Supplementary Figure 10: details of UDP donor substrate interactions in the LH3 GT domain. (A) Co-crystallization using Mn^{2+} yields clear electron density for the metal ion in the GT domain ($2F_o$-F_c omit electron density maps, green mesh, contour level 1.2 σ). This density is located near metal ion-coordinating residues Asp112, Asp115 and His253. (B) Using UDP-Gal or UDP-Glc donor substrates, additional electron density is found near the pyrophosphate of the UDP. Shown is the $2F_o$-F_c omit electron density maps (purple mesh, contour level 1.0 σ) in the glycan binding site of LH3 co-crystallized in presence of UDP-Gal. A possible conformation of the UDP-Gal substrate, inferred from structures of UDP-Gal-bound structures available in the protein data bank, is shown with black sticks (UDP-Gal model from PDB 5MD7). The poly-Asp sequence 187-191 is shown with green sticks. (C) 2D representation of the interaction network surrounding the UDP molecules found in UDP-Gal and UDP-Glc co-crystal structures. Dashed lines indicate H bonds, red crowns highlight hydrophobic interactions. This image was created using LIGPLOT+[10].

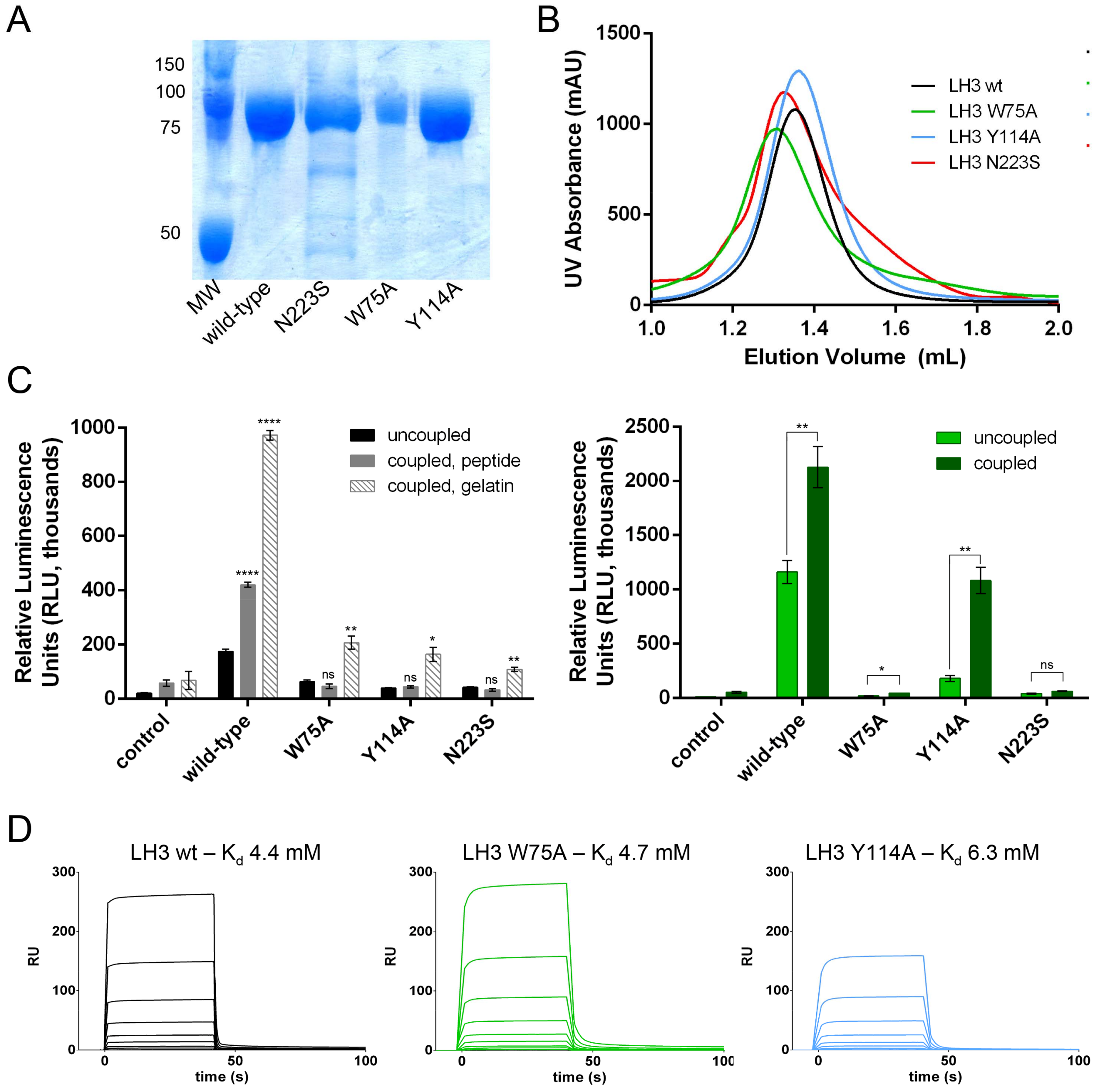

Supplementary Figure 11: biochemical characterization of LH3 mutants in the GT domain. (A) SDS-PAGE analysis of recombinant wild-type and mutant LH3 enzymes. (B) Size-exclusion chromatograms showing that all mutants generated consistently elute at retention times comparable to wild-type LH3. (C) Mutations in the GT domain affect LH enzymatic activity. The histograms show the relative luminescence associated to LH (left) and GGT (right) activities of wild-type and mutant LH3. For LH assays, we used the ARGIKGIRGFS peptide. Error bars and statistical evaluations as in Figure 2 and Supplementary Fig. 1. (D) SPR measurement of binding of the ARGIKGIRGFS synthetic peptide to wild type and mutant LH3. Concentration ranges were between 8 μM and 2 mM in all experiments. Mutant N223S could not be measured due to low immobilization on the SPR chip. Kd values were calculated at the steady-state using the Biacore T200 evaluation software (GE Healthcare).

```
              293         303         313         323         333
Human_LH3       QPPPRVFLAV  FVEQPTPFLP  RFLQRLLLLD  YPPDRVTLFL  HNNEVFHEPH
Human_LH1       EALPTVLVGV  FIEQPTPFVS  LFFQRLLRLH  YPQKHMRLFI  HNHEQHHKAQ
Human_LH2       DVHPNVSIGV  FIEQPTPFLP  RFLDILLTLD  YPKEALKLFI  HNKEVYHEKD
consensus       :  * * :.*  *:******:.  *:: ** *    ** . : **:  **:* .*:

              343         353         363         373         383
Human_LH3       IADSWPQLQD  HFSAVKLVGP  EEALSPGEAR  DMAMDLCRQD  PECEFYFSLD
Human_LH1       VEEFLAQHGS  EYQSVKLVGP  EVRMANADAR  NMGADLCRQD  RSCTYYFSVD
Human_LH2       IKVFFDKAKH  EIKTIKIVGP  EENLSQAEAR  NMGMDFCRQD  EKCDYYFSVD
consensus       :     :       .::*:***  *  :: .:**  :*. *:****   .* :***:*

              393         403         413         423         433
Human_LH3       ADAVLTNLQT  LRILIEENRK  VIAPMLSRHG  KLWSNFWGAL  SPDEYYARSE
Human_LH1       ADVALTEPNS  LRLLIQQNKN  VIAPLMTRHG  RLWSNFWGAL  SADGYYARSE
Human_LH2       ADVVLTNPRT  LKILIEQNRK  IIAPLVTRHG  KLWSNFWGAL  SPDGYYARSE
consensus       **..**: .:  *.:**::*.:  :***:::***  .*********  *.* ******

              443         453         463         473         483
Human_LH3       DYVELVQRKR  VGVWNVPYIS  QAYVIRGDTL  RMELPQRDVF  SGSDTDPDMA
Human_LH1       DYVDIVQGRR  VGVWNVPYIS  NIYLIKGSAL  RGELQSSDLF  HHSKLDPDMA
Human_LH2       DYVDIVQGNR  VGVWNVPYMA  NVYLIKGKTL  RSEMNERNYF  VRDKLDPDMA
consensus       ***::** .*  ********::  : *:*.*.:*  * *: . : *    .. *****

              493         503         513
Human_LH3       FCKSFRDKGI  FLHLSNQHEF  GRLLATSR
Human_LH1       FCANIRQQDV  FMFLTNRHTL  GHLLSLDS
Human_LH2       LCRNAREMGV  FMYISNRHEF  GRLLSTAN
Consensus       :* . *: .:  *:.::*.* :  *.**:
```

Supplementary Figure 12: sequence alignment between AC domains of human LH isoforms. Residues matching those involved in Mn^{2+} coordination in related glycosyltransferases are shown with pink triangles. Disulfide bonds are shown with connecting lines. Consensus symbols defined as in Supplementary Fig. 9.

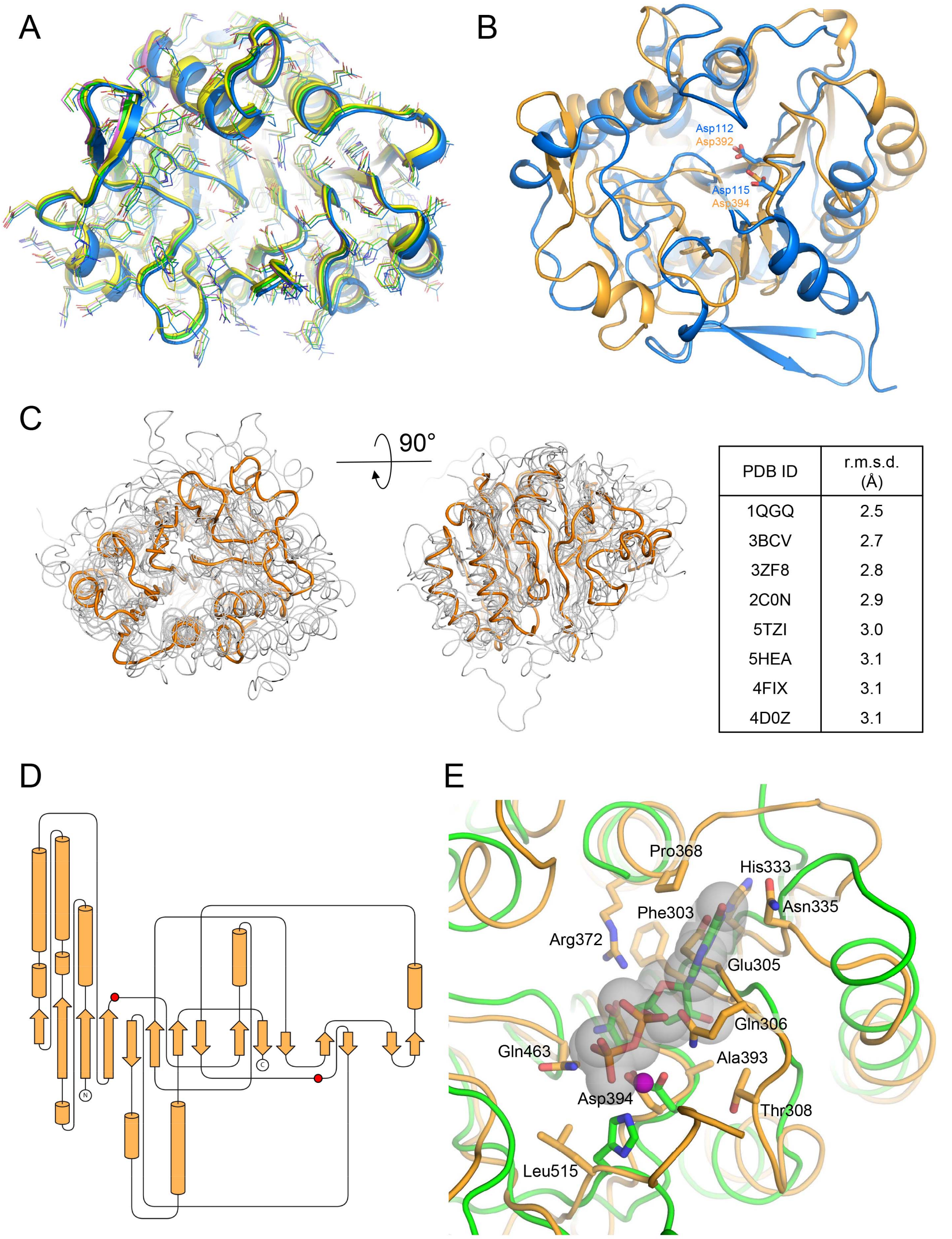

PDB ID	r.m.s.d. (Å)
1QGQ	2.5
3BCV	2.7
3ZF8	2.8
2C0N	2.9
5TZI	3.0
5HEA	3.1
4FIX	3.1
4D0Z	3.1

Supplementary Figure 13: structural features of the AC domain. (A) Superposition of ligand-free (blue) and ligand-bound LH3 structures does not show differences in the AC domain. (B) Superposition of the GT (blue) and AC (orange) domains of LH3. Residues involved in Mn^{2+} binding in the GT domain, and the corresponding homologous residues in the AC domain, are shown as sticks. (C) Superposition of the LH3 AC domain (thick orange ribbon) with related glycosyltransferase structures as found by DALI[7] and PDBeFold[8] (thin grey ribbon). (D) Topology diagram of the LH3 AC domain. Residues Asp392 and Asp394 are shown with red circles. (E) Modeling of donor substrates in the AC domain highlights steric clashes. Shown is the superposition of the LH3 AC domain (orange) with the structure of *Saccharomyces cerevisiae* Mnn9 bound to GDP (green, PDB ID 3ZF8)[11] shows that, although the metal ion binding site (purple sphere) is preserved, the region hosting the ligand cannot accommodate any UDP-like molecules (grey spheres) because of steric hindrance with multiple LH3 residues (orange sticks).

.

A

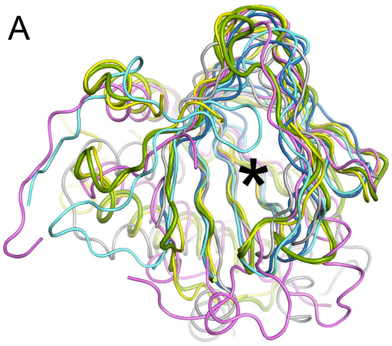

PDB ID	% id.	r.m.s.d. (Å)
6AX7	41	1.3
4ZUA	9	2.0
5IAV	21	2.2
5UQP	12	2.2
4MV2	12	2.3

B

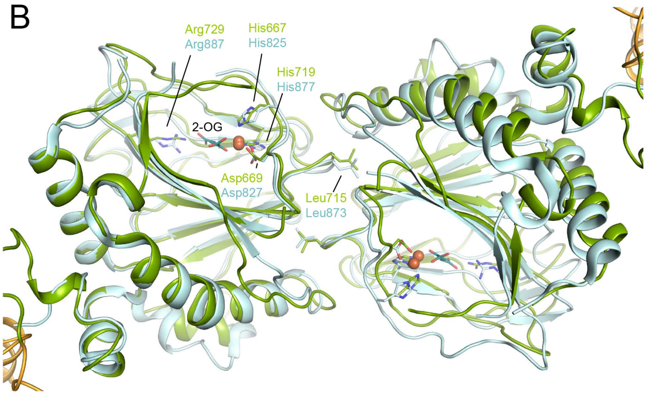

C

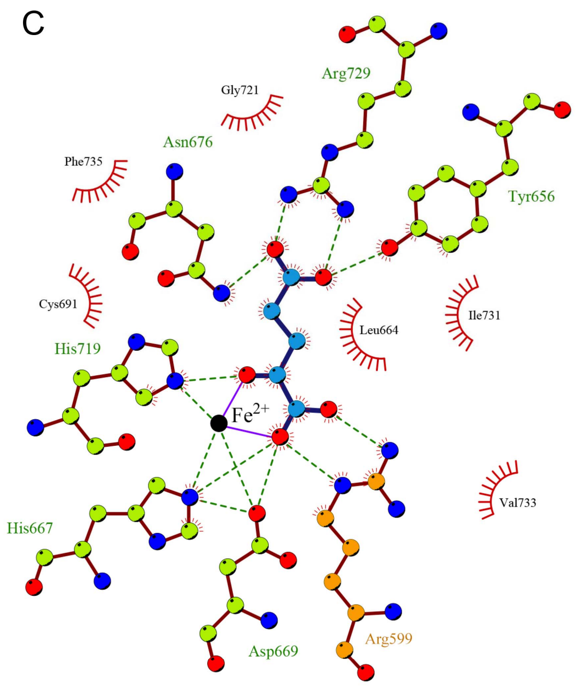

Supplementary Figure 14: features of the LH3 LH domain. (A) Superposition with homologous Fe^{2+}, 2-OG dioxygenases identified using DALI[7] and PDBeFold[8]. LH3 is shown in dark grey. The location of the Fe^{2+} and 2-OG cofactors is shown with a black asterisk. (B) Superposition of the LH3 LH domain (green) with the recently reported crystal structure of the LH domain fragment of viral homolog L230 (cyan). Cofactors are represented as in Figure 3. (C) 2D representation of the interaction network surrounding the 2-OG cofactor in the LH catalytic site. Arg599 involved in metal ion-induced stabilization is shown in orange. Dashed lines indicate H bonds, red crowns highlight hydrophobic interactions. This image was created using LIGPLOT+[10].

```
                521         531         541         551         561
Human_LH3       YDTEHLHPDL  WQIFDNPVDW  KEQYIHENYS  RALEGEGIVE  QPCPDVYWFP
Human_LH1       YRTTHLHNDL  WEVFSNPEDW  KEKYIHQNYT  KALAGK-LVE  TPCPDVYWFP
Human_LH2       YNTSHYNNDL  WQIFENPVDW  KEKYINRDYS  KIFTEN-IVE  QPCPDVFWFP
consensus       * * * : **  *::*.** **  **:**: :*:  . :  : :**   *****:***

                571         581         591         601         611
Human_LH3       LLSEQMCDEL  VAEMEHYGQW  SGGRHEDSRL  AGGYENVPTV  DIHMKQVGYE
Human_LH1       IFTEVACDEL  VEEMEHFGQW  SLGNNKDNRI  QGGYENVPTI  DIHMNQIGFE
Human_LH2       IFSEKACDEL  VEEMEHYGKW  SGGKHHDSRI  SGGYENVPTD  DIHMKQVDLE
consensus       :::*  ****  * ****:*:*  * *.: *.*:   ********   ****:*:. *

                621         631         641         651         661
Human_LH3       DQWLQLLRTY  VGPMTESLFP  GYHTKARAVM  NFVVRYRPDE  QPSLRPHHDS
Human_LH1       REWHKFLLEY  IAPMTEKLYP  GYYTRAQFDL  AFVVRYKPDE  QPSLMPHHDA
Human_LH2       NVWLHFIREF  IAPVTLKVFA  GYYTKGFALL  NFVVKYSPER  QRSLRPHHDA
consensus         * :::  :  :.*:* .::.  **:*..   :   ***.* *:   * ** ****:

                671         681         691         701         711
Human_LH3       STFTLNVALN  HKGLDYEGGG  CRFLRYDCVI  SSPRKGWALL  HPGRLTHYHE
Human_LH1       STFTINIALN  RVGVDYEGGG  CRFLRYNCSI  RAPRKGWTLM  HPGRLTHYHE
Human_LH2       STFTINIALN  NVGEDFQGGG  CKFLRYNCSI  ESPRKGWSFM  HPGRLTHLHE
consensus       ****:*:***  . * *::***  *.****:* *   :*****:::  ******* **

                721         731
Human_LH3       GLPTTWGTRY  IMVSFVDP
Human_LH1       GLPTTRGTRY  IAVSFVDP
Human_LH2       GLPVKNGTRY  IAVSFIDP
consensus       ***...****  * ***:**
```

Supplementary Figure 15: sequence alignment showing LH domains of human LH isoforms. Residues involved in catalytic Fe^{2+} coordination are shown with orange triangles, residues stabilizing the 2-OG co-substrate are shown with light blue triangles. Residue Leu715 is shown in dark blue. Residues constituting the flexible capping loop are shown in red with yellow background. Residues coordinating the second Fe^{2+} in metal ion-inhibited structures are shown in magenta. Residue Arg599, mimicking the lysine substrate in the stabilized conformations induced by metal ion binding is shown with a green triangle. Disulfide bonds are shown with connecting lines. Consensus symbols defined as in Supplementary Fig. 9.

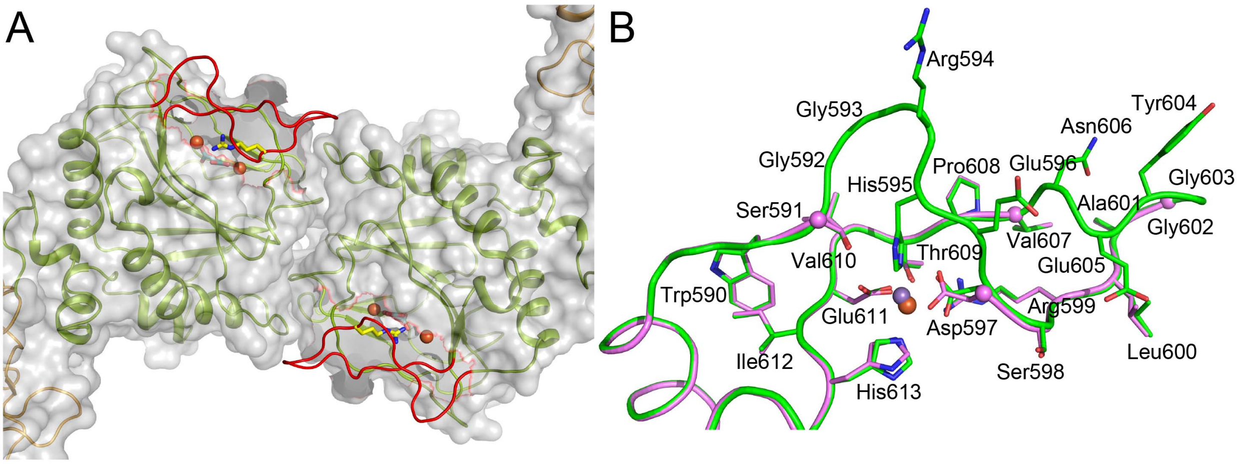

Supplementary Figure 16: excess metal ion binding induces a stabilized conformation of the LH domain. (A) The flexible capping loop 590-610 (red ribbon with Arg599 shown with yellow sticks) stabilized by additional metal ions in the LH domain is close to the LH-LH dimer interface. Shown is the semi-transparent surface of the LH domain, highlighting the deep groove at the dimer interface. (B) Superposition between Fe^{2+}-bound (green) and Mn^{2+}-bound (pink) LH3 loop 590-610, with highlight of amino acid side chains surrounding the metal ion binding site. Although with increased flexibility (as highlighted by lack of density and associated molecular model for amino acids 592-596 and 603-605, model boundaries shown with pink spheres), the Mn^{2+}-bound structure shows a very similar arrangement of this capping loop.

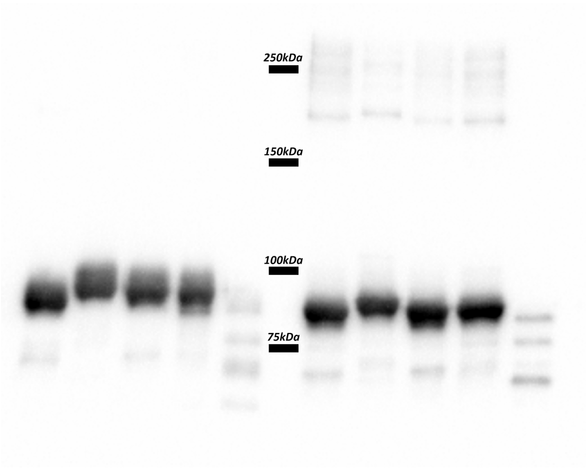

Supplementary Figure 17: uncropped image of the western blot shown in Supplementary Figure 7B. Molecular weight markers are indicated in central lane.

Supplementary Table 1: crystallographic statistics for data collection, structure solution and refinement.

	LH3	LH3 + Mn^{2+}	LH3 + Fe^{2+} + Mn^{2+} + UDP-Gal	LH3 + Fe^{2+} + Mn^{2+} + UDP-Glc	LH3 + Fe^{2+} + Mn^{2+} + UDP-Gal (Hg^{2+} soak)	LH3 + Fe^{2+} + Mn^{2+} + UDP-Gal (native SAD)
**Data Collection[a]**						
X-ray source	SLS X06SA	ESRF ID30A-3	ESRF ID30A-3	ESRF ID30A-3	ESRF ID30B	SLS X06DA
Processing programs	XDS, AIMLESS	XDS, AIMLESS	XDS, AIMLESS	XDS, AIMLESS	XDS, AIMLESS	AUTOPROC/ STARANISO,XSCALE
Space group	$C222_1$	$C222_1$	$C222_1$	$C222_1$	$C222_1$	$C222_1$
Cell parameters	a = 97.3 Å; α = 90° b = 100.1 Å; β = 90° c = 226.4 Å; γ = 90°	a = 97.2 Å; α = 90° b = 100.0 Å; β = 90° c = 225.1 Å; γ = 90°	a = 97.8 Å; α = 90° b = 100.3 Å; β = 90° c = 224.9 Å; γ = 90°	a = 97.7 Å; α = 90° b = 100.5 Å; β = 90° c = 225.4 Å; γ = 90°	a = 98.7 Å; α = 90° b = 100.3 Å; β = 90° c = 226.0 Å; γ = 90°	a = 97.1 Å; α = 90° b = 100.0 Å; β = 90° c = 224.3 Å; γ = 90°
Wavelength (Å)	1.0000	0.9677	0.9677	0.9677	1.0059	2.0751
Resolution (Å)	69.78-2.70 (2.83-2.70)	48.79-2.10 (2.15-2.10)	49.78-2.10 (2.15-2.10)	48.87-2.50 (2.60-2.50)	49.34-3.00 (3.18-3.00)	112.4-2.14 (2.31-2.14)
Total reflections	99584 (12906)	499999 (35541)	340513 (25017)	196048 (22780)	539022 (83344)	30842446 (3001260)
Unique reflections	30019 (3967)	64193 (4455)	63999 (4484)	38604 (4301)	22895 (3654)	56666 (9637)
CC1/2[b]	0.990 (0.435)	0.999 (0.658)	0.997 (0.582)	0.996 (0.497)	0.999 (0.958)	0.998 (0.739)
Redundancy	3.3 (3.3)	7.8 (8.0)	5.3 (5.6)	5.1 (5.3)	23.5 (22.8)	544.3 (311.4)
Mean I/σ(I)	4.6 (0.7)	12.2 (0.9)	11.0 (1.2)	8.7 (0.9)	13.9 (3.9)	18.8 (2.4)
Completeness (%)	98.0 (98.5)	99.8 (99.8)	98.8 (99.7)	99.6 (99.7)	99.9 (99.9)	93.5 (78.35)
R_{sym}[c]	0.166 (1.309)	0.095 (2.444)	0.117 (1.335)	0.143 (1.590)	0.208 (0.849)	n.d.
R_{pim}[d]	0.146 (1.163)	0.054 (1.389)	0.081 (0.922)	0.103 (1.147)	0.060 (0.251)	0.054 (0.086)

[a] Values in parentheses are for reflections in the highest resolution shell.

[b] Resolution limits were determined by applying a cut-off based on the mean intensity correlation coefficient of half-datasets (CC1/2) approximately of 0.5 [12], , except for data processed with AUTOPROC/STARANISO.

[c] $R_{sym} = [\ \Sigma_{hkl}\Sigma_j\ |\ I_{hkl,j} - <I_{hkl}>\ |\ ]\ /\ [\ \Sigma_{hkl}\Sigma_j\ I_{hkl,j}\ ]$, where I is the observed intensity for a reflection and <I> is the average intensity obtained from multiple observations of symmetry-related reflections.

[d] $R_{pim} = [\ \Sigma_{hkl}\ (1/(n-1))^{1/2}\ \Sigma_j\ |\ I_{hkl,j} - <I_{hkl}>\ |\ ]\ /\ [\ \Sigma_{hkl}\Sigma_j\ I_{hkl,j}\ ]$ where I is the observed intensity for a reflection and <I> is the average intensity obtained from multiple observations of symmetry-related reflections.

	LH3	LH3 + Mn^{2+}	LH3 + Fe^{2+} + Mn^{2+} + UDP-Gal	LH3 + Fe^{2+} + Mn^{2+} + UDP-Glc	LH3 + Fe^{2+} + Mn^{2+} + UDP-Gal (Hg^{2+} soak)	LH3 + Fe^{2+} + Mn^{2+} + UDP-Gal (native SAD)
**Refinement**						
Heavy atom sites					3[c]	
Molecules per ASU	1	1	1	1	1	1
R_{work}/R_{free}[d]	0.278/0.309	0.229/0.252	0.218/0.236	0.216/0.248	0.238/0.265	0.194/0.231
Average B-factor (Å)^2	66.5	50.9	48.5	62.3	73.6	33.7
Protein	66.3	50.6	48.3	62.1	73.6	33.4
Ligands	76.5	74.7	66.8	75.2	83.5	49.9
Solvent		49.5	46.6	63.2		34.9
Number of atoms:	5556	5914	6191	5997	5810	6150
Protein	5491	5572	5754	5754	5721	5762
Ligands	65	79	92	92	89	92
Solvent		263	345	151		296
**Structure quality**						
RMS bond lengths (Å)	0.002	0.003	0.003	0.002	0.002	0.004
RMS bond angles (°)	0.48	0.62	0.65	0.59	0.50	0.70
**Ramachandran stats**						
Favored (%)	95	96	97	96	95	96
allowed (%)	5	4	3	4	5	4
outliers (%)	0	0	0	0	0	0
PDB ID	6FXK	6FXM	6FXR	6FXT	6FXX	6FXY

[c] Number of heavy atom sites refers to those additional to the Fe^{2+} and Mn^{2+} already found in other LH3 structures.

[d] R_{free} values are calculated based on 5% randomly selected reflections.

Supplementary Table 2A: list of disease-related mutations identified in LH enzymes and associated biochemical features

Mutation(s)	Corresponding residue(s) in LH3	observed features		Disease phenotype	notes	References
		LH activity	GT/GGT activities			
LH1 Gln49*	Glu60	n/a	n/a	Ehlers-Danlos Syndrome Type VI		Yeowell et al., 2000[13]
LH1 Leu85Pro	Met97	n/a	n/a	Ehlers-Danlos Syndrome Type VI		Rohrbach et al., 2011[14]
LH1Tyr142*	Tyr154	n/a	n/a	Ehlers-Danlos Syndrome Type VI		Yeowell et al., 2000[15]
LH2a Ser166* (LH2b Ser166*)	Ser166	n/a	n/a	kyphomelic dysplasia		Leal et al., 2018[16]
LH3 Asn223Ser	Asn223	+/-	-/-	Connective tissue disorder (similar to osteogenesis imperfecta)	Introduces glycosylation near UDP-donor substrate binding site	Salo et al., 2008[17] This work
LH1 Arg319*	Thr329	n/a	n/a	Ehlers-Danlos Syndrome Type VI		Hyland et al., 1992[18] Eyre et al., 2002[19] Giunta et al., 2005[20]
LH1Gln327*	Gln316	n/a	n/a	Ehlers-Danlos Syndrome Type VI		Yeowell et al., 2000[15]
LH1 Gln345*	Ser355	n/a	n/a	Ehlers-Danlos Syndrome Type VI		Rohrbach et al., 2011[14]
LH1 Δ367-371	Asp377 Leu378 Cys379 Arg380 Gln381	-	n/a	Ehlers-Danlos Syndrome Type VI		Yeowell et al., 2000[13]
LH2a Arg380Cys (LH2b Arg380Cys)	Arg380	n/a	n/a	Bruck Syndrome Type II		Lv et al., 2018[21]
LH2a Cys385Arg (LH2b Cys385Arg)	Cys385	n/a	n/a	Bruck Syndrome Type II		Lv et al., 2018[21]
LH1 Trp419Leu*	Trp429	n/a	n/a	Ehlers-Danlos Syndrome Type VI		Rohrbach et al., 2011[14]
LH1 Trp446Gly	Trp456	n/a	n/a	Ehlers-Danlos Syndrome Type VI		Walker et al., 2005[22]
LH1 Ile454*	Ala464	n/a	n/a	Ehlers-Danlos Syndrome Type VI		Giunta et al., 2005[20]

LH1 Tyr 511*	Tyr521	+	n/a	Ehlers-Danlos Syndrome Type VI		Walker et al., 1999[23] Yeowell et al., 2000[13,15]
LH1 Δ532	Glu542	n/a	n/a	Ehlers-Danlos Syndrome Type VI		Ha et al., 1994[24]
LH2a Trp540* (LH2b Trp561*)	Trp540	n/a	n/a	skeletal dysplasia Kozlowski-Reardon		Leal et al., 2018[16]
LH2a Asp564Val (LH2b Asp585Val)	Asp565	n/a	n/a	kyphomelic dysplasia	Near LH-LH dimerization interface, abolishes H-bond with conserved residues	Leal et al., 2018[16]
LH2a Trp567Cys (LH2b Trp588Cys)	Trp568	n/a	n/a	moderate osteogenesis imperfecta		Leal et al., 2018[16]
LH2a Arg598His (LH2b Arg619)	Arg599	-	n/a	Bruck Syndrome Type II	Forms a complex with 2-OG mimicking substrate Lys in metal ion-stabilized structures	Ha-Vinh et al., 2004[25] Hyry et al., 2009[26]
LH2a Gly601Cys (LH2b Gly622)	Gly602	-	n/a	Bruck Syndrome Type II	Near LH-LH dimerization interface in substrate mimicry structures	Van der Slot et al., 2003 Hyry et al., 2009[26] Puig-Hervas et al., 2012
LH2a Gly601Val (LH2b Gly622)	Gly602	-	n/a	Bruck Syndrome Type II	Near LH-LH dimerization interface in substrate mimicry structures	Van der Slot et al., 2003[27] Hyry et al., 2009[26] Puig-Hervas et al., 2012[28]
LH2a Thr608Ile (LH2b Thr629)	Thr609	-	n/a	Bruck Syndrome Type II	Near Arg599 in substrate mimicry structures 10-fold decreased affinity for 2-OG	Van der Slot et al., 2003[27] Hyry et al., 2009[26] Puig-Hervas et al., 2012[28] Leal et al., 2018[16]
LH1 Trp612Cys	Trp623	n/a	n/a	Ehlers-Danlos Syndrome Type VI		Brinckmann et al., 1998[29]
LH2a Gly640Asp LH2b Gly661Asp	Gly641	n/a	n/a	Bruck Syndrome Type II		Lv et al., 2018[21]

LH2a His666Arg (LH2b His687Arg)	His667	n/a	n/a	Bruck Syndrome Type II	Coordinates Fe^{2+} in LH catalytic site	Leal et al., 2018[16]
LH1 Ala667Thr	Ala678	n/a	n/a	Ehlers-Danlos Syndrome Type VI		Giunta et al., 2005[20]
LH1 Arg670*	His681	n/a	n/a	Ehlers-Danlos Syndrome Type VI		Yeowell et al., 2000[15]
LH1 Gly678Arg	Gly689	n/a	n/a	Ehlers-Danlos Syndrome Type VI		Ha et al., 1994[24]<br>Yeowell et al., 2000[13]<br>Rohrbach et al., 2011[14]
LH2a Arg659* (LH2b Arg680*)	Glu660	n/a	n/a	Bruck Syndrome Type II		Lv et al., 2018[21]
LH3 Cys691Ala*	Cys691	-	++	Connective tissue disorder (similar to osteogenesis imperfecta)		Salo et al., 2008[17]
LH1 His706Arg	His717	n/a	n/a	Ehlers-Danlos Syndrome Type VI	Near LH-LH dimerization interface	Giunta et al., 2005[20]

Supplementary Table 2B: list of biochemical mutations used to characterize LH structure and function

Mutation(s)	Corresponding residue(s) in LH3	observed features: LH activity	observed features: GT/GGT activities	notes	References
LH3 Trp75Ala	Trp75	+/-	-	Interacts with UDP	This work
LH1 His88Ser	Tyr100	+	n/a		Pirskanen et al., 1996[30]
LH3 Tyr114Ala	Tyr114	+/-	+/-	Interacts with UDP	This work
LH3 Leu136V	Leu136	n/a	+++		Wang et al., 2002[31]
LH3 Phe143Leu	Phe143	n/a	+		Wang et al., 2002[31]
LH3 Cys144Ile	Cys144	++	-		Wang et al., 2002[31]
LH3 Trp148Asn-Leu150Thr	Trp148Asn-Leu150Thr	++	+		This work
LH1 Asn163Gln	Thr175	++	n/a		Pirskanen et al., 1996[30]
LH3 Asp187Ala, Asp189Ala	Asp187, Asp189	n/a	-		Wang et al., 2002[31]
LH3 Asp187Ala, Asp189Ala, Asp190Ala, Asp191Ala	Asp187, Asp189, Asp190, Asp191	n/a	-		Wang et al., 2002[31]
LH1 Asn197Gln	Ser209	+	n/a		Pirskanen et al., 1996[30]
LH3 Leu208Ile	Leu208	n/a	++		Wang et al., 2002[31]
LH1 His225Ser	Arg238	++	n/a		Pirskanen et al., 1996[30]
LH1 His241Ser	His253	+	n/a	Coordinates Mn^{2+} in GT catalytic site	Pirskanen et al., 1996[30]
LH3 Asp392Ala	Asp392	n/a	++		Wang et al., 2002[31]
LH3 Ala464Ile	Ala464	n/a	++		Wang et al., 2002[31]
LH1 His474Ser	Gly484	+	n/a		Pirskanen et al., 1996[30]
LH1 Asp491Ala	Gly501	+++	n/a		Pirskanen et al., 1996[30]
LH1 His517Ser	His527	+	n/a		Pirskanen et al., 1996[30]
LH1 His536Ser	His546	+	n/a		Pirskanen et al., 1996[30]

LH1 Asn538Gln	Asn548	++	n/a	Glycosylation site	Pirskanen et al., 1996[30]
LH3 Met560Leu	Met560	n/a	++		Wang et al., 2002[31]
LH1 His613Ser	Leu624	+	n/a		Pirskanen et al., 1996[30]
L230 Lys804Glu	His643	+	n/a		Guo et al., 2018[6]
LH1 Asp638Ala	Val649	+	n/a		Pirskanen et al., 1996[30]
LH1 Asp648Ala	Asp659	+	n/a		Pirskanen et al., 1996[30]
LH1 His656Ser	His667	-	n/a	Coordinates Fe^{2+} in LH catalytic site	Pirskanen et al., 1996[30]
L230 His825Ala	His667	-	n/a	Coordinates Fe^{2+} in LH catalytic site	Guo et al., 2018[6]
LH1 His657Ser	His668	+	n/a		Pirskanen et al., 1996[30]
LH3 Asp669Ala	Asp669	-	n/a	Coordinates Fe^{2+} in LH catalytic site	Heikkinen et al., 2000[32]
L230 Asp827Ala	Asp669	-	n/a	Coordinates Fe^{2+} in LH catalytic site	Guo et al., 2018[6]
LH1 Asp658Ala	Asp669	-	n/a	Coordinates Fe^{2+} in LH catalytic site	Pirskanen et al., 1996[30]
LH1 Asp647Ala	Asp685	+	n/a		Pirskanen et al., 1996[30]
LH3 Thr672Asn	Thr672	-	+/-	Involved in the LH-LH dimerization interface	This work
LH1 Asn686Gln	Asp696	+	n/a		Pirskanen et al., 1996[30]
LH1 His700Ser	His711	+	n/a		Pirskanen et al., 1996[30]
L230 Leu873Asp	Leu715	-	n/a	Involved in the LH-LH dimerization interface	Guo et al., 2018[6]
LH3 Arg714Asn	Arg714	-	++	Involved in the LH-LH dimerization interface	This work
LH3 Leu715Asp	Leu715	++	++	Involved in the LH-LH dimerization interface	This work
LH3 Leu715Arg	Leu715	-	+	Involved in the LH-LH dimerization interface	This work
LH1 His706Ser	His717	++	n/a		Pirskanen et al., 1996[30]
L230 His825Ala	His719	-	n/a	Coordinates Fe^{2+} in LH catalytic site	Guo et al., 2018[6]
LH1 His708Ser	His719	+++	n/a	Coordinates Fe^{2+} in LH catalytic site	Pirskanen et al., 1996[30]

L230 Ala879Gly	Gly721	++	n/a		Guo et al., 2018[6]
LH1 Arg715Ala	Trp726	+	n/a		Passoja et al., 1998[33]
L230 Arg886Ala	Arg729	-	n/a	Stabilizes 2-OG in LH catalytic site	Guo et al., 2018[6]
LH1 Arg718Ala	Arg729	-	n/a	Stabilizes 2-OG in LH catalytic site<br>10-fold decreased affinity for 2-oxo-glutarate	Passoja et al., 1998[33]
LH3 1-231<br>(deletion construct)		-	-		Heikkinen et al., 2000[32]
LH3 Δ283-297		n/a	+	Use of specific antibodies to block this region	Heikkinen et al., 2000[32]
LH3 33-520<br>(deletion construct)		n/a	+		Wang et al., 2002[34]
LH3 33-401<br>(deletion construct)		n/a	+		Wang et al., 2002[34]
LH3 33-388<br>(deletion construct)		n/a	-		Wang et al., 2002[34]
LH3 1-521<br>(deletion construct)		-	+		Heikkinen et al., 2000[32]
LH3 1-668<br>(deletion construct)		-	+		Heikkinen et al., 2000[32]

Supplementary Table 3: summary of SAXS data analysis.

	SEC-SAXS	Batch Measurements						
		0.6 mg/mL	1.1 mg/mL	2.0 mg/mL	2.5 mg/mL	3.4 mg/mL	4.2 mg/mL	9.0 mg/mL
**Data Collection**								
Beamline	ESRF BM29	ESRF BM29	ESRF BM29	ESRF BM29	ESRF BM29	ESRF BM29	ESRF BM29	ESRF BM29
Beam energy (keV)	12.5	12.5	12.5	12.5	12.5	12.5	12.5	12.5
Sample-detector distance (m)	2.867	2.867	2.867	2.867	2.867	2.867	2.867	2.867
Exposure time (s)	1	1	1	1	1	1	1	1
Sample cell thickness (mm)	1	1	1	1	1	1	1	1
Temperature (°)	20	20	20	20	20	20	20	20
Final q range (nm^{-1})	0.01 - 4	0.01 - 4	0.01 - 4	0.01 - 4	0.01 - 4	0.01 - 4	0.01 - 4	0.01 - 4
**Data Analysis**								
Points used for Guinier analysis	9-43	7-41	9-36	12-27	9-28	11-23	9-23	11-21
Guinier qR_g limits	1.25	1.25	1.19	1.08	1.16	1.13	1.17	1.18
Guinier R_g (nm)	5.05	6.9	7.3	8.3	8.9	9.5	10.1	10.9
I(0) (mm^{-1})	61.5 ± 0.1	255.7 ± 0.8	296.2 ± 0.8	381.4 ± 0.8	390.0 ± 0.9	631.3 ± 1.7	613.9 ± 2.0	517.8 ± 1.5
D_{max} (nm)	21.0	23.5	30.9	31.5	36.1	40.9	47.1	48.6
MW estimation (V_c based) (kDa)	182	247	286	338	390	489	500	569

Supplementary Table 4: list of cloning and mutagenesis primers used in this study

Construct name	Vector	Sense	Oligo sequence (5'-3')	5' enzyme	3' enzyme
LH3	pUPE.106.08; pPuro-DHFR	Fw	ggatccTCCGACCGGCCCCGGGGC	BamHI	NotI
LH3	pUPE.106.08; pPuro-DHFR	Rv	gcggccgcGGGGTCGACAAAGGACACCATGAT	BamHI	NotI
LH3-W75A	pUPE.106.08	Fw	gcgCGAGGGGGTGATGTGGCTC	BamHI	NotI
LH3-W75A	pUPE.106.08	Rv	CTCCTCTCCCAGGCCCAGGGT	BamHI	NotI
Y114A	pUPE.106.08	Fw	gcgGACGTGATTCTGGCCGGC	BamHI	NotI
Y114A	pUPE.106.08	Rv	GCTATCCACAAACATGATGATCATATCC	BamHI	NotI
LH3-W148N-L150T	pUPE.106.08	Fw	aatGGcacGGCGGAGCAGTACC	BamHI	NotI
LH3-W148N-L150T	pUPE.106.08	Rv	CTCGGGCCAGCAGAAGCTCTC	BamHI	NotI
LH3-N223S	pUPE.106.08	Fw	tccGGGGCTTTAGATGAAGTGGTT	BamHI	NotI
LH3-N223S	pUPE.106.08	Rv	GGTTCTGAAAGATCCGAGACTTATG	BamHI	NotI
LH3-T672N	pUPE.106.08	Fw	aCTTCACCCTCAACGTTGCCCTCAAC	BamHI	NotI
LH3-T672N	pUPE.106.08	Rv	TGGATGAGTCGTGGTGTGGCCGC	BamHI	NotI
LH3-R714N	pUPE.106.08	Fw	aaCCTCACCCACTACCACGAGGG	BamHI	NotI
LH3-R714N	pUPE.106.08	Rv	GCCGGGGTGCAGGAGTGCC	BamHI	NotI
LH3-L715D	pUPE.106.08	Fw	gacACCCACTACCACGAGGG	BamHI	NotI
LH3-L715D	pUPE.106.08	Rv	GCGGCCGGGGTGCAGGAGT	BamHI	NotI
LH3-L715R	pUPE.106.08	Fw	agaACCCACTAC CACGAGGGG	BamHI	NotI
LH3-L715R	pUPE.106.08	Rv	GCGGCCGGGGTGCAGGAGT	BamHI	NotI

SUPPLEMENTARY REFERENCES

1. Evans, P.R. & Murshudov, G.N. How good are my data and what is the resolution? *Acta Crystallogr D Biol Crystallogr* **69**, 1204-14 (2013).
2. Vonrhein, C. et al. Data processing and analysis with the autoPROC toolbox. *Acta Crystallographica Section D* **67**, 293-302 (2011).
3. Krissinel, E. & Henrick, K. Inference of macromolecular assemblies from crystalline state. *J Mol Biol* **372**, 774-97 (2007).
4. Svergun, D., Barberato, C. & Koch, M.H.J. CRYSOL - a Program to Evaluate X-ray Solution Scattering of Biological Macromolecules from Atomic Coordinates. *Journal of Applied Crystallography* **28**, 768-773 (1995).
5. Konarev, P.V., Volkov, V.V., Sokolova, A.V., Koch, M.H.J. & Svergun, D.I. PRIMUS: a Windows PC-based system for small-angle scattering data analysis. *Journal of Applied Crystallography* **36**, 1277-1282 (2003).
6. Guo, H.F. et al. Pro-metastatic collagen lysyl hydroxylase dimer assemblies stabilized by Fe(2+)-binding. *Nat Commun* **9**, 512 (2018).
7. Holm, L. & Rosenstrom, P. Dali server: conservation mapping in 3D. *Nucleic Acids Res* **38**, W545-9 (2010).
8. Krissinel, E. & Henrick, K. Secondary-structure matching (SSM), a new tool for fast protein structure alignment in three dimensions. *Acta Crystallogr D Biol Crystallogr* **60**, 2256-68 (2004).
9. Bond, C.S. TopDraw: a sketchpad for protein structure topology cartoons. *Bioinformatics* **19**, 311-2 (2003).
10. Laskowski, R.A. & Swindells, M.B. LigPlot+: multiple ligand-protein interaction diagrams for drug discovery. *J Chem Inf Model* **51**, 2778-86 (2011).
11. Striebeck, A., Robinson, D.A., Schuttelkopf, A.W. & van Aalten, D.M. Yeast Mnn9 is both a priming glycosyltransferase and an allosteric activator of mannan biosynthesis. *Open Biol* **3**, 130022 (2013).
12. Karplus, P.A. & Diederichs, K. Linking crystallographic model and data quality. *Science* **336**, 1030-3 (2012).
13. Yeowell, H.N. & Walker, L.C. Mutations in the lysyl hydroxylase 1 gene that result in enzyme deficiency and the clinical phenotype of Ehlers-Danlos syndrome type VI. *Mol Genet Metab* **71**, 212-24 (2000).
14. Rohrbach, M. et al. Phenotypic variability of the kyphoscoliotic type of Ehlers-Danlos syndrome (EDS VIA): clinical, molecular and biochemical delineation. *Orphanet J Rare Dis* **6**, 46 (2011).
15. Yeowell, H.N., Walker, L.C., Farmer, B., Heikkinen, J. & Myllyla, R. Mutational analysis of the lysyl hydroxylase 1 gene (PLOD) in six unrelated patients with Ehlers-Danlos syndrome type VI: prenatal exclusion of this disorder in one family. *Hum Mutat* **16**, 90 (2000).
16. Leal, G.F. et al. Expanding the Clinical Spectrum of Phenotypes Caused by Pathogenic Variants in PLOD2. *J Bone Miner Res* **33**, 753-760 (2018).

17. Salo, A.M. et al. A connective tissue disorder caused by mutations of the lysyl hydroxylase 3 gene. *Am J Hum Genet* **83**, 495-503 (2008).
18. Hyland, J. et al. A homozygous stop codon in the lysyl hydroxylase gene in two siblings with Ehlers-Danlos syndrome type VI. *Nat Genet* **2**, 228-31 (1992).
19. Eyre, D., Shao, P., Weis, M.A. & Steinmann, B. The kyphoscoliotic type of Ehlers-Danlos syndrome (type VI): differential effects on the hydroxylation of lysine in collagens I and II revealed by analysis of cross-linked telopeptides from urine. *Mol Genet Metab* **76**, 211-6 (2002).
20. Giunta, C., Randolph, A. & Steinmann, B. Mutation analysis of the PLOD1 gene: an efficient multistep approach to the molecular diagnosis of the kyphoscoliotic type of Ehlers-Danlos syndrome (EDS VIA). *Mol Genet Metab* **86**, 269-76 (2005).
21. Lv, F. et al. Novel Mutations in PLOD2 Cause Rare Bruck Syndrome. *Calcif Tissue Int* **102**, 296-309 (2018).
22. Walker, L.C. et al. A novel mutation in the lysyl hydroxylase 1 gene causes decreased lysyl hydroxylase activity in an Ehlers-Danlos VIA patient. *J Invest Dermatol* **124**, 914-8 (2005).
23. Walker, L.C., Marini, J.C., Grange, D.K., Filie, J. & Yeowell, H.N. A patient with Ehlers-Danlos syndrome type VI is homozygous for a premature termination codon in exon 14 of the lysyl hydroxylase 1 gene. *Mol Genet Metab* **67**, 74-82 (1999).
24. Ha, V.T., Marshall, M.K., Elsas, L.J., Pinnell, S.R. & Yeowell, H.N. A patient with Ehlers-Danlos syndrome type VI is a compound heterozygote for mutations in the lysyl hydroxylase gene. *J Clin Invest* **93**, 1716-21 (1994).
25. Ha-Vinh, R. et al. Phenotypic and molecular characterization of Bruck syndrome (osteogenesis imperfecta with contractures of the large joints) caused by a recessive mutation in PLOD2. *Am J Med Genet A* **131**, 115-20 (2004).
26. Hyry, M., Lantto, J. & Myllyharju, J. Missense mutations that cause Bruck syndrome affect enzymatic activity, folding, and oligomerization of lysyl hydroxylase 2. *J Biol Chem* **284**, 30917-24 (2009).
27. van der Slot, A.J. et al. Identification of PLOD2 as telopeptide lysyl hydroxylase, an important enzyme in fibrosis. *J Biol Chem* **278**, 40967-72 (2003).
28. Puig-Hervas, M.T. et al. Mutations in PLOD2 cause autosomal-recessive connective tissue disorders within the Bruck syndrome--osteogenesis imperfecta phenotypic spectrum. *Hum Mutat* **33**, 1444-9 (2012).
29. Brinckmann, J. et al. Ehlers-Danlos syndrome type VI: lysyl hydroxylase deficiency due to a novel point mutation (W612C). *Arch Dermatol Res* **290**, 181-6 (1998).
30. Pirskanen, A., Kaimio, A.M., Myllyla, R. & Kivirikko, K.I. Site-directed mutagenesis of human lysyl hydroxylase expressed in insect cells. Identification of histidine residues and an aspartic acid residue critical for catalytic activity. *J Biol Chem* **271**, 9398-402 (1996).
31. Wang, C. et al. The third activity for lysyl hydroxylase 3: galactosylation of hydroxylysyl residues in collagens in vitro. *Matrix Biol* **21**, 559-66 (2002).

32. Heikkinen, J. et al. Lysyl hydroxylase 3 is a multifunctional protein possessing collagen glucosyltransferase activity. *J Biol Chem* **275**, 36158-63 (2000).
33. Passoja, K., Rautavuoma, K., Ala-Kokko, L., Kosonen, T. & Kivirikko, K.I. Cloning and characterization of a third human lysyl hydroxylase isoform. *Proc Natl Acad Sci U S A* **95**, 10482-6 (1998).
34. Wang, C. et al. Identification of amino acids important for the catalytic activity of the collagen glucosyltransferase associated with the multifunctional lysyl hydroxylase 3 (LH3). *J Biol Chem* **277**, 18568-73 (2002).
